# Fourth annual workshop on fetal alcohol spectrum disorders prevention and clinical guidelines research: co-occurring conditions

**DOI:** 10.1186/s12919-026-00386-0

**Published:** 2026-07-24

**Authors:** Tracey Pérez Koehlmoos, Elizabeth H. Lee, Ilse Rivera, Jennifer Wisdahl, Tom Donaldson

**Affiliations:** 1 Center for Health Services Research, Uniformed Services University of the Health Sciences, 4301 Jones Bridge Road, Bethesda, US; 2 Department of Pediatrics, Uniformed Services University of the Health Sciences, 4301 Jones Bridge Road, Bethesda, US; 3 The Henry M. Jackson Foundation for the Advancement of Military Medicine, 6720A Rockledge Dr, Bethesda, US; 4 FASD United, 1054 31st Street NW #204, Washington, DC US

## Executive summary

Fetal alcohol spectrum disorders (FASDs) are a group of neurodevelopmental disorders that result from prenatal exposure to alcohol. These disorders can include a variety of developmental, behavioral, intellectual and physical challenges during childhood and throughout the lifespan. FASD is among the leading preventable causes of developmental disabilities in the United States, with important implications for the health and well-being of military service members and their families as well as the general American public. 

Addressing FASD in the context of the U.S. military requires tailored strategies to support individuals and their families, with a focus on prevention, early intervention, and comprehensive care within the U.S. Department of War (DoW) Military Health System (MHS). Lessons emerging from these efforts can also help to improve care for FASD-affected families in the broader civilian population. 

The Uniformed Services University of the Health Sciences (USUHS), in conjunction with FASD United, hosted the fourth annual *Workshop on Fetal Alcohol Spectrum Disorders Prevention and Clinical Guidelines Research* on 17 September 2025 in Washington, DC. As part of the FASD Prevention and Clinical Guidelines Research Initiative, a four-year, federally funded research program, the workshop provided a forum for exchanging research findings and discussing opportunities to create care approaches that are grounded in, and adaptable to, the latest insights. 

Over the course of the workshop, speakers reported on the initiative's progress, reviewed current knowledge and practices, and identified potential strategies to enhance prevention, screening, diagnosis, interventions, and family support. Nearly 100 attendees from academia, health care, federal agencies, and patient advocacy organizations participated in discussions to reflect on research outcomes, share perspectives on the experiences of families and the clinicians who care for them, and identify opportunities to better meet the needs of children, adults, and families affected by FASD.

## Background

The FASD Prevention and Clinical Guidelines Research Initiative is a four-year, federally funded project initiated in 2022 that is focused on FASD in the MHS. As part of the initiative, the Center for Health Services Research (CHSR) at USUHS, FASD United, and the Boston University School of Public Health are working together to examine the experiences of families and providers in the military community who care for and support individuals with FASD.

The project’s primary aim is to increase awareness of and response to FASD and its risk factors within the MHS. In addition, by strengthening the research basis for FASD prevention and clinical guidelines, researchers and practitioners involved in the project hope to improve care delivery to military children and families and create a new model of FASD care delivery that could be replicated in the civilian sector.

As one of the nation’s largest and most comprehensive healthcare systems, the MHS delivers direct services and private-sector care to approximately 9.6 million beneficiaries, including active-duty service members, military retirees, and their families. The CHSR directly supports the DoW and MHS, conducts research aligned with MHS strategic goals, provides Health Services Research (HSR) training for students and faculty, and collaborates with civilian HSR organizations. FASD United is a leading hub for the FASD community that seeks to expand the reach of FASD-informed diagnostic, medical, behavioral health, and non-clinical services while also advocating for policy changes that ensure full inclusion in all care systems and benefits programs for individuals with FASD. FASD United also works to prevent prenatal exposure to alcohol and other harmful substances.

## Introduction

USUHS and FASD United hosted the *4th Annual Workshop on Fetal Alcohol Spectrum Disorders Prevention and Clinical Guidelines Research* on 17 September 2025 in Washington, DC. Nearly 100 attendees from academia, health care, federal agencies, and patient advocacy organizations gathered to review the latest research and discuss key issues surrounding FASD during the day-long workshop (see Appendix A for a list of attendees).

The event was the fourth in a series of workshops held as part of the FASD Prevention and Clinical Guidelines Research Initiative (“FASD Project”). The first of these annual workshops was held in 2022 and provided an overview of the existing research, programs, and clinical practice guidelines focused on prenatal alcohol exposure (PAE) and FASD [1]. The second workshop, held in 2023, detailed several lines of effort (LoEs), including plans for assessments of community needs and population health, the use of electronic health records and predictive analytics to improve early diagnosis and patient outcomes, and a hub-and-spoke model for telehealth [2]. The third workshop, held in 2024, focused on ways to advance support, interventions, and therapies for both families and children [3]. The 2025 workshop, summarized in this report, examined results from multiple studies that have been completed to date under this initiative, provided an update on work that is still in progress, and offered a forum for discussing opportunities to address remaining gaps in research and care delivery.

USUHS’s external partners in this effort are FASD United, Boston University School of Public Health, and the Henry M. Jackson Foundation for the Advancement of Military Medicine; internal USUHS partners are the CHSR; the School of Medicine Departments of Preventive Medicine & Biostatistics, Clinical Psychology, Gynecologic Surgery and Obstetrics, Family Medicine, and Pediatrics; and the Graduate School of Nursing.

The workshop presentations and discussions centered around children and families living with FASD while also navigating other co-occurring neurobehavioral and physical conditions. In some studies, PAE and Fetal Alcohol Syndrome (FAS) are used as proxies for FASD, which is often underdiagnosed or not reflected in formal diagnostic codes and datasets.

Tracey Pérez Koehlmoos, Director of the CHSR at USUHS, and Tom Donaldson, FASD United President and CEO, provided welcoming remarks. They expressed optimism that the novel effort represented by the FASD Project and the findings presented at the workshop could be transformative for the FASD community, as well as for the community of patients, families, advocates, clinicians, and researchers interested in neurodevelopmental disorders more broadly. A theme throughout the workshop was the co-occurrence of other disorders—behavioral, psychological, and physical—alongside FASD. The workshop began with a keynote presentation that highlighted the risk of adverse mental health behaviors, in particular suicide, among people with disabilities, which may include people with FASD. Next, researchers presented key findings and future directions emerging from several LoEs undertaken as part of the FASD Project. These research presentations were followed by a panel on the experiences and perspectives of individuals with FASD.

Throughout the workshop, participants emphasized the critical need to improve screening, diagnosis, interventions, and care coordination for individuals and families. To do this effectively, they highlighted the importance of addressing stigmas, recognizing the whole-body effects of FASD and the multiple co-occurring conditions experienced by many people with FASD, and creating effective training, supports, and systems of care to enable clinicians to better serve patients and families for the best possible outcomes.

## Bridging data and prevention in adverse mental health behaviors

Nicole Marlow, PhD, MSPH, University of Florida College of Public Health and Health Professions, opened the workshop with a keynote address on the complex factors that influence a person’s risk of adverse mental health behaviors, specifically those related to suicide. She underscored the importance of embedding suicide prevention in programs for people with neurodevelopmental disorders and described emerging research findings that can help to inform these suicide prevention efforts.

### The social ecological model of public health

To frame her remarks, Marlow described the social ecological model of public health, which captures how factors at multiple levels influence health outcomes. This model reflects the fact that each person exists within multiple spheres which can influence health in different ways. A core tenet of this framework is that health is influenced not only by biology or individual characteristics, but also by environmental and social factors. At the individual level, health can be influenced by knowledge, attitudes, skills, and behaviors. The interpersonal level reflects the influence of friends, family, and social networks. The institutional level includes the organizations, schools, and workplaces; the community level includes cities, neighborhoods, resources, and norms; and the policy level encompasses federal, state, and local legislation.

Researchers have sought to identify how factors within these different spheres influence the risk of suicide. Marlow described the high prevalence of suicide, which accounted for over 49,000 deaths in the United States in 2023, equating to one death every 11 min. In addition to those who die by suicide, millions of U.S. adults suffer from suicidal thoughts, engage in suicide planning, or attempt suicide each year. To find opportunities to support individuals and prevent suicide, she emphasized the importance of considering how multiple factors at multiple levels can influence suicide risk for individuals and communities. For example, researchers have examined associations between suicide risk and factors such as access to mental health services, cultural stigmas around mental health, and policies such as gun laws and legislation related to mental health care. Even physical environmental factors such as altitude may influence risk.

### Suicide risk among people with disabilities

Marlow described three recent studies examining patterns in suicidal behaviors among people with disabilities. The studies are based on data from over 200,000 individuals who participated in the National Survey on Drug Use and Health between 2015–2019. In this work, the presence or absence of disabilities was determined by participants self-reporting their functional limitations using six questions that originated with the U.S. Census. While this method does not provide granular data about particular medical diagnoses, it broadly captures how different types of limitations—such as limitations affecting hearing, vision, cognition, physical ability—affect a person’s daily life and ability to live independently.

Marlow noted that people who self-identified as having disabilities also shared several other characteristics. Compared with study participants without disabilities these individuals tended to be older and were more likely to be female, less likely to belong to a minority racial or ethnic group, and less likely to live in a metropolitan area. They were also more often single, more often unemployed, and had lower average educational attainment and lower average household income. Finally, people with disabilities were more likely to have other chronic conditions, have fair or poor health, visit hospital emergency rooms, and experience episodes of major depression.

Compared to people without disabilities, Marlow and colleagues found that people with disabilities had more than double the risk of suicidal behaviors, including a 2.13-fold higher risk of suicidal ideation, 2.66-fold higher risk of suicide planning, and 2.47-fold higher risk for a suicide attempt [4]. A separate study examined these risks among people with different types of disabilities, finding that people with cognitive limitations (defined as having serious difficulty concentrating, remembering, or making decisions) or complex activity limitations (which make it difficult to perform daily activities such as dressing, bathing, or doing errands alone) showed the highest risk [5]. This study also revealed that people with more limitations had a significantly elevated risk compared with those who had fewer limitations.

A third study examined the interactions between veteran status and disability status in terms of the risk of suicidal behaviors [6]. Previous studies have shown that both veterans and people with disabilities face a higher risk of suicide compared with the general population. However, this study showed that veterans with a disability actually had a lower risk for suicidal behaviors compared with non-veterans with a disability. Veterans showed an elevated risk compared with non-veterans only among people without disability and only for suicide planning, not for suicidal ideation or suicide attempts. Marlow suggested that suicide prevention efforts focused on veterans and the Veterans Administration’s centralized model for mental health care could account for the generally lower risk seen among veterans with disabilities compared with individuals with disabilities who are not veterans.

### Future directions

Next, Marlow aims to expand on this work with a new study focused on suicide risk and prevention among people with neurodevelopmental disorders, which would be especially relevant for the FASD population and military families. She described a proposed research study that would look at individual, community, and regulatory factors that may influence suicidal behaviors among people with neurodevelopmental disorders, including a granular examination of outcomes among people with different types of disorders. Preliminary data collected to inform this proposal indicate that the prevalence of suicidal behaviors has been rising since before the COVID-19 pandemic and that the growth has been especially pronounced among people with neurodevelopmental disorders relative to those without such disorders, underscoring the urgency of finding effective interventions to address suicide risk. In the meantime, she emphasized that all programs for the neurodevelopmental disorders community should incorporate strategies to increase access to mental health support and prevent suicide.

## FASD prevention and clinical guidelines research project updates

In the workshop’s second session, researchers shared emerging findings from projects related to the FASD Prevention and Clinical Guidelines Research Initiative (“the FASD Project”). To open the session, Elizabeth H. Lee, DrPH, of USUHS Pediatrics and the USUHS CHSR, and Sarah Brown, MPH, of FASD United, shared outcomes of the first three years of activities within the FASD Project. Then, investigators involved in the ongoing LoEs within the initiative shared emerging findings and insights from their work over the past year. Throughout the session, presenters highlighted cross-cutting issues and themes related to scientific practices and progress on FASD.

### FASD Project efforts and outcomes to date

Lee noted that the FASD Project is supported by Congressionally-directed funding with an overarching goal to increase awareness and response to FASD and its risk factors within the MHS. While its primary focus is on improving care delivery to military children and families affected by FASD, the project also aims to create a model of FASD care delivery that may be replicated among national healthcare systems to benefit the general population. Lee noted that the project applies to children who may or may not have a formal diagnosis of FASD.

**Fig. 1:**
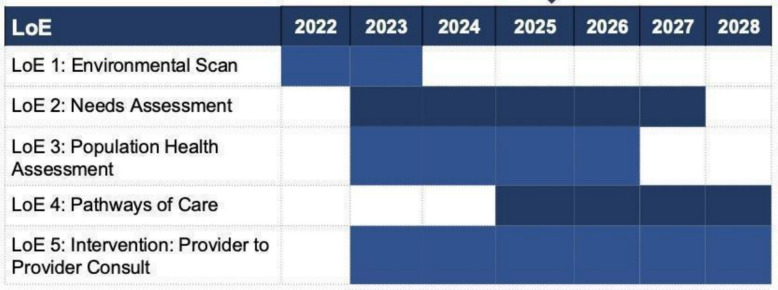
Timelines of activity within each of the five lines of effort for the FASD Prevention and Clinical Guidelines Research initiative. Source [7]: Lee EH. (2025). 5 Lines of Effort in FASD Prevention and Clinical Guidelines Project in Review: Years 1–3 [PowerPoint slides]. FASD Annual Workshop

The FASD Project is overseen by USUHS CHSR and conducted in partnership with FASD United, Boston University, and Madigan Army Medical Center. 2025 marks the end of the project’s third year, and project activities are slated to continue through 2028. Lee described outcomes to date along with ongoing activities across the project’s five LoEs (Fig. 1).

The first LoE is complete and involved an environmental scan to understand the continuum of care for FASD across the MHS [8].

The second LoE is a community needs assessment. This effort is in progress and includes multiple components. The first is underway, and is a set of in-depth interviews with healthcare providers in the MHS who care for children and women of reproductive age. Additional interviews with caregivers of children with FASD, as well as women who are currently pregnant or were recently pregnant (up to 24 months post-partum) are planned through the end of 2027.

The third LoE is a population health assessment focusing on identifying the population of children with FASD in the MHS, using diagnostic codes for FAS and PAE as a proxy. The first component of this effort, focusing on children with a diagnosis, is now complete [9]. A second component is underway that aims to identify the ‘shadow’ population of children without a diagnosis who may have key risk factors such as diagnoses of co-occurring neurodevelopmental disorders.

The fourth LoE is an analysis of pathways of care, which aims to understand the services children with FASD and co-occurring neurodevelopmental disorders access as they move through the MHS. This effort will begin concurrent with the third LoE and identification of the undiagnosed shadow population.

The fifth LoE, still underway, aims to develop a provider-to-provider consult intervention in order to shorten the timeline from screening to diagnosis for children in the MHS. An initial review has been published, which provides a foundation for understanding the effectiveness of interventions that have been previously studied in this space [10].

Two additional components were undertaken as part of this project and are nearly complete, with results to be reported in forthcoming manuscripts. These include a survey of family physicians’ knowledge and practice related to FASD, and a separate survey focused on providers’ attitudes toward adoption of the Survey of Well-Being of Young Children (SWYC). The SWYC is a validated screening tool for developmental assessment, which is the required screener in use at military hospitals and clinics.

### Resources developed by FASD United

Brown highlighted an array of resources FASD United has developed as part of its activities as a partner for the FASD Project.

One key outcome is the Language and Stigma Guide, a 60-page, comprehensive guide for talking about PAE and FASD in accurate and non-stigmatizing language. Intended for individuals, organizations, and professionals across many spheres, the guide provides recommendations for using person-first language, debunking stereotypes, and fostering a culture of inclusion. The guide was viewed over 2,500 times in the first 10 months following its publication in December 2024; a Spanish translation is slated for release in late 2025.

FASD United also hosts regular events to foster exchange among FASD stakeholders including researchers, practitioners, and family advocates. These include research symposia and an annual international research conference. White papers capturing themes from the data shared at these events provide a resource to help move the field forward [11].

Brown also described how the organization gathers data and conducts studies to identify needs and gaps among the FASD community. The National Education Survey is one such effort. FASD United conducts this survey semiannually to quantify the challenges students with FASD face at school and identify needs, barriers, and strengths in the education space [12]. In another effort, FASD United collects and summarizes state-level data to capture how FASD is being addressed within each state. These data reflect how each state’s Department of Health recognizes, tracks, and communicates about alcohol exposure during pregnancy and FASD outcomes and services, as well as summarizing state-level rates of unplanned pregnancies and binge drinking [13]. Another data-gathering effort that is underway is a diagnostic capacity assessment, which aims to understand FASD diagnostic capacity at the national level to identify key barriers along with opportunities to expand access.

Finally, FASD United serves families affected by PAE through its Affiliate Network and its Family Navigator Program. The FASD United Affiliate Network is a group of 48 autonomous organizations that work to advance FASD awareness, provide support, and improve quality of life for those affected by PAE. Through the Family Navigator Program, people who have FASD or are caregivers for people with FASD serve as navigators to provide families with one-on-one peer support, referrals, and assistance with accessing benefits and services.

### Provider experiences with FASD in the MHS

Ilse Rivera, MPH, of the CHSR at USUHS, shared emerging insights from interviews with healthcare providers being conducted as part of the FASD Project’s second LoE, the needs assessment.

Previous research suggests there is hesitancy to diagnose FASD among some providers due to two main factors: a lack of confidence stemming from the lack of standard diagnostic criteria, and a fear of exposing children and families to stigma. To better understand how these and other factors may influence FASD care in the MHS, Rivera and colleagues designed a study using qualitative interviews to examine attitudes and knowledge among MHS providers who care for children. A separate study will examine experiences providing care related to alcohol use or alcohol use disorders among MHS clinicians caring for women of reproductive age.

This research focuses specifically on providers at military hospitals and clinics who care for TRICARE beneficiaries. To date, Rivera and colleagues have conducted 13 qualitative interviews with MHS providers caring for children in these settings. Although this research is still ongoing, with a planned total of 25–30 interviews, Rivera shared preliminary insights that have emerged from the initial set of interviews.

When asked about their experiences assessing children for FASD and PAE, participating providers indicated that they use the SWYC, the Ages and Stages Questionnaire, and their own observations and those of caregivers in assessing early development. Some reported relying on the physical presentation of FAS, specifically facial features, when deciding whether to consider FASD. Even when FASD is possible, some providers noted that they are concerned that raising the topic can risk undermining trust or “burning bridges” with families, jeopardizing the provider-family relationship. Participants articulated a need for concise, easily accessible resources to help guide FASD assessment.

Participants’ perspectives on FASD diagnosis echoed those in previous studies, Rivera said. Specifically, the study results reported to date provide further evidence that the lack of diagnostic criteria undermines providers’ confidence in diagnosing FASD, and that the potential to bring stigma to the family also contributes to a reluctance to diagnose FASD. In the specific context of the MHS, she noted that providers were also aware that the impacts of this stigma could extend to effects on the service career of the parent or the potential future service career of the child.

Providers also highlighted the importance of a multidisciplinary approach to clinical management for children with FASD. Children may need a variety of interventions and services, such as behavioral pediatrics or speech therapy, but many families face challenges in accessing these services, whether through the MHS directly or through private civilian facilities. Wait times to receive services can range from several months up to a year. Participants expressed enthusiasm for telehealth-based provider-to-provider consults as a potential opportunity to help address this gap.

When asked about their knowledge and education related to FASD, most participants reported little to no exposure to FASD education beyond what they received in medical school and residency. They expressed a desire for more education on the topic, but identified time as a limiting factor, reiterating the need for condensed, accessible, FASD-specific resources for providers in order to increase FASD awareness and help guide conversations with families with strategies to maintain patient trust and avoid stigma.

Going forward, Rivera and colleagues will continue to recruit providers to participate in interviews, including those caring for women of reproductive age. Future studies will focus on in-depth interviews with caregivers of children with FASD and with women who are pregnant or were recently pregnant (up to 24 months post-partum) to understand the living experiences of these different groups as they navigate care within the MHS.

### FASD and overlapping conditions in military children

Madison Cirillo, MPH, of the CHSR at USUHS, described findings related to the FASD Project’s third LoE, a population health assessment focused on understanding the scope of FASD, both diagnosed and undiagnosed, within the MHS. Her presentation highlighted studies of FASD and overlapping neurodevelopmental conditions in military children based on secondary data analysis using the MHS Data Repository.

For context, she noted that the prevalence of neurodevelopmental disorders has increased in recent years. According to the National Health Interview Study, the proportion of U.S. children with any diagnosed developmental disability (which includes intellectual disabilities (ID), autism spectrum disorder (ASD), and other developmental delays) rose from 7.4% to 8.6% between 2019–2021 [14].

Among this group, children with FASD face unique challenges and often have other overlapping conditions, with the most common being attention-deficit/hyperactivity disorder (ADHD). Research on the co-occurrence of FASD and ASD is more limited, although in one small study, 68% of 99 patients with FASD were found to have ASD or social communication disorder [15].

To understand the prevalence of FASD among children in the MHS, Cirillo and colleagues analyzed military-connected children who have a diagnosis of FAS or PAE as a proxy for FASD, which has no diagnosis code. They found 1,476 children with FAS, PAE, or both in their health records between fiscal years 2016–2023, reflecting an estimated prevalence of 0.42 cases per 1,000 for that period. On average, these children received their first diagnosis at age 8, although the average age of PAE diagnosis was lower at age 4 and the average age of FAS diagnosis was higher at age 10 [9].

Researchers then conducted a follow-on study to assess rates of co-occurring neurodevelopmental disorders among the same patient group. This study revealed that 84.6% of children with an FAS or PAE diagnosis in the MHS had one or more of the following additional neurodevelopmental disorders: ADHD, ID, ASD, communication disorders, delayed development, motor disorders, specific learning disorders, tic disorders, or other neurodevelopmental disorders. Nearly half (46.6%) had three or more neurodevelopmental disorder diagnoses in addition to FASD, suggesting that many of these children are likely to need significant and comprehensive treatment and services, Cirillo said.

Consistent with previous research, the study revealed that the most common co-occurring conditions with FASD were ADHD, ID, and ASD. Co-occurring neurodevelopmental disorders were more common among boys than girls overall, and a higher proportion of White children had at least one co-occurring neurodevelopmental disorder compared with Black children. Analyses of specific neurodevelopmental disorders revealed similar patterns in the prevalence of particular disorders by biological sex and race, and researchers also examined associations between the co-occurrence of neurodevelopmental disorders and the rank and branch of the child’s military sponsor. For example, findings suggest that having a sponsor in the Marine Corps or a Junior enlisted sponsor decreases the likelihood of having a co-occurring neurodevelopmental disorder. A vast majority (93.9%) of children who were living in guardianship or in a pre-adoptive ward or foster home had at least one co-occurring neurodevelopmental disorder.

Based on insights from these studies, Cirillo suggested that the strong co-occurrence of various neurodevelopmental disorders along with PAE and FAS could help aid in the identification of children with FASD. The researchers plan further analyses to identify additional related conditions and risk factors to inform strategies to identify at-risk children for further screening.

### Co-occurrence of neurodevelopmental, mental health, and alcohol and substance use disorders

Elizabeth Hisle-Gorman, PhD, USUHS Pediatrics, shared her work focused on understanding the relationships between neurodevelopmental disorders, mental health disorders, and alcohol and substance use disorders.

Existing research on alcohol and substance use and neurodevelopmental disorders, such as ADHD and ASD, shows conflicting data regarding the risk of alcohol and substance use and the potential impact of stimulant medications (used for ADHD) on that risk. In the case of ASD, studies suggest that the risk of alcohol and substance use disorders may vary in accordance with a person’s age and level of social impairment.

Research more clearly establishes the connections between alcohol and substance use disorders and mental health conditions, especially anxiety, depression, suicidal ideation, schizophrenia, and personality disorders.

To investigate these relationships within the Military Health System (MHS), Hisle-Gorman and colleagues analyzed diagnoses from the 2018–2023 MHS data repository. This study population included children of active-duty or retired service members, as well as young adults ages serving in the military, 0 to 25 years of age.

Of the approximately 4.8 million eligible individuals, approximately 1.7% (*n* = 82,151) and 2.7% (131,442) were diagnosed with alcohol use disorder, or either alcohol use disorder or substance use disorder (AUD/SUD), respectively. One-quarter of the study population (1,182,996) had a mental health condition, and of those, nearly 5% (53,235) had an alcohol use disorder and 8% (89,908) had an AUD/SUD. Neurodevelopmental disorders were present in 12% (598,486) of the eligible study population. Among those individuals, just over 1% (7,421) and 3% (17,955) had an alcohol use disorder or an AUD/SUD, respectively.

The prevalence of mental health conditions was considerably higher in individuals with an alcohol use disorder 64% (52,577) or an AUD/SUD 68% (89,381) drawn from the overall study population. The prevalence of neurodevelopmental disorders was 9% (7,394) in individuals with an alcohol use disorder (lower than the overall population prevalence) but was 15% (19,848) in those with an AUD/SUD (higher than the overall population prevalence).

The prevalence of alcohol use disorder and AUD/SUD differed when stratified by age (children 0–17 years; young adults 18–25 years which included service members and older children of service members). Alcohol use disorder and AUD/SUD were present in 0.2% and 1% of children, respectively. This was increased in young adults at 3.2% and 4.5%, respectively. Alcohol use disorder and AUD/SUD was significantly more common (both > 80%) in children with mental health diagnoses compared to those without a mental health diagnosis (25%, *p* < 0.05). In young adults, a similar pattern was evident, with alcohol use disorder and AUD/SUD present in > 60% of those with a mental health diagnosis compared to those without (23%, *p* < 0.05).

Approximately 40% of children with a neurodevelopmental disorder had either an alcohol use disorder or AUD/SUD diagnosis, compared to 20% of those without a neurodevelopmental diagnosis (*p* < 0.05). In young adults, 5.8% of those with a neurodevelopmental disorder were also diagnosed with alcohol use disorder, and another 10.7% were diagnosed with an AUD/SUD.

Based on these findings, Hisle-Gorman suggested that focusing on early identification and intervention for mental health and developmental diagnoses should be an important component of any strategy to prevent alcohol and substance use disorders among young people.

### Disability, alcohol use, and addiction

Rachel Sayko Adams, PhD, MPH, Boston University School of Public Health, spoke about the intersections among disability, alcohol use, and addiction. Her presentation drew from work conducted under the Intersecting Research on Opioid and Alcohol Misuse, Addiction, and Disability Services (INROADS) studies, which focus on understanding how people with disabilities are affected by alcohol use, opioid use, and addiction [16]. As part of those studies, researchers have examined subgroups of people with disabilities, including those with intellectual and developmental disabilities, with relevance to the FASD community.

On the whole, Adams said that research has shown that people with disabilities experience both structural and interpersonal barriers as well as health differences that impact their care. They often have greater physical and mental health needs than the general population but experience discrimination and a range of barriers in accessing quality healthcare overall, a pattern which also applies to the specific context of services related to substance use and addiction. People with disabilities are also subject to stereotypes and incorrect assumptions about substance use and may be unfairly deemed noncompliant if they are unable to engage in standard treatment protocols or too complex to receive evidence-based addiction medications. These issues can reinforce the “othering” of people with disabilities and undermine providers’ recognition of their obligation to offer person-centered care that includes accommodations, Adams said.

To understand these concerns and identify opportunities to improve access to quality substance use disorder treatment among people with disabilities, Adams and colleagues investigated patterns in alcohol use in this population. Using data from the Behavioral Risk Factor Surveillance Survey, the team found that people with disabilities were less likely to report using alcohol and engage in binge drinking on the whole compared with people who did not have disabilities. However, among people who reported currently using alcohol, people with disabilities were more likely to engage in binge drinking than those without disabilities, suggesting that people with disabilities who drink alcohol may do so in riskier ways [17]. Studies drawing from other datasets have shown similar patterns, suggesting that people with disabilities may face a greater risk for high-intensity drinking, alcohol use disorder, and addiction [18, 19].

The evidence base for understanding associations between FASD and substance use disorders is quite limited. Several studies have suggested that people with FASD encounter a higher risk of alcohol or substance use problems as they age. One study reported alcohol or drug problems in 46% of adults 21 or older with FASD and 29% of youth aged 12–20 [20], while another reported alcohol use disorders in 25% of 21-year-old adults with FASD [21]. In these studies, older age at diagnosis, a less stable or nurturing home, and exposure to maternal alcohol consumption earlier in pregnancy were found to be predictors of these problems. However, Adams noted that available studies on this topic tend to be older, have small sample sizes, and lack validated instruments to assess substance use.

The research base is more robust when examining substance use among people with intellectual and developmental disabilities more broadly [22]. While many people with these disabilities do not drink alcohol or use drugs, studies suggest that those who do use alcohol or substances appear to have a higher rate of substance misuse. Adams pointed to several studies showing increased rates of substance use disorders among people with ASD or ADHD, with the highest rates seen among individuals who have both conditions. Given this context and the frequent co-occurrence of FASD with both ADHD and ASD, Adams underscored the need for more research, in particular longitudinal studies, to understand the effects of FASD on alcohol and other substance use throughout the lifespan [22].

Adams also pointed to an urgent need to improve access to effective treatments for substance use disorders among people with disabilities broadly, who face barriers as a result of stigma, provider hesitancy, inaccessible materials, and other factors. While studies have clearly documented treatment differences among people with disabilities in the context of opioid use disorder, less is known about treatment experiences for these individuals in the context of alcohol use disorders. One study showed that people with disabilities were almost twice as likely to report an unmet need for substance use disorder treatment compared with adults without a disability [23]. In another study, researchers conducted a systematic review and found that there is a lack of interventions for people with FASD directly targeting mental health and substance use challenges [24].

Emphasizing that successful treatment is possible, Adams said that it is essential to work with people with disabilities to understand how best to adapt treatment approaches to accommodate them. She added that providers should not assume that only people with visible disabilities have needs or expect people with disabilities to take on the burden of ensuring they receive accommodations. For additional guidance on the treatment of substance use disorders in people with disabilities, she pointed to a 2019 advisory from the Substance Abuse and Mental Health Services Administration [25] and a chapter on addressing cognitive impairment in substance use disorder treatment in the 2023 edition of the American Society of Addiction Medicine Criteria [26].

### Toward a model of provider-to-provider consultation

Eric Flake, MD, Col USAF (ret) of the CHSR at USUHS, shared research and activities in support of a framework for providing identification, evaluation, diagnostic, and intervention services for FASD-impacted individuals and families that is based on provider-to-provider consultation, the FASD Project’s fifth LoE. Noting that the mental health field has made INROADS toward provider-to-provider consultation models for improving access to care, Flake outlined the rationale for developing similar models to improve clinical neurodevelopmental support for military-connected children, who navigate a variety of services and move through multiple systems of care.

To understand why it is important to enhance clinical care for children with FASD, it is illustrative to examine the evidence base for interventions that are designed to increase positive behaviors or reduce maladaptive or negative behaviors among these children. Toward this end, Flake and colleagues conducted a review of reviews providing evidence on behavioral interventions targeting areas such as executive function, self-management, social skills, and behavioral regulation [10]. Specifically, the researchers examined studies of child-centered, non-pharmacological interventions taking place outside of a school setting. Contrary to historical notions that brain-based neurodevelopmental conditions cannot be changed or improved, these studies provide robust evidence that direct, child-centered interventions can lead to positive impacts on executive function and social skills.

Although the review focused on child-centered interventions, Flake noted that these interventions can also work synergistically with family-centered interventions. Based on the study findings, the researchers posit that reinforcement of regular, structured interventions can enhance the potential for long-term developmental gains. For future work, they point to opportunities to further integrate children into interventions and extend the length of interventions to build upon established foundations.

To inform approaches to improve care delivery through provider-to-provider consultation, Flake highlighted the importance of recognizing the variety of comorbidities and manifestations that are associated with FASD [27]. These include neurodevelopmental and learning issues such as ADHD, learning disabilities, and speech and language issues, as Cirillo discussed. They also include psychiatric and behavioral issues such as sleep disorders, mood disorders, and substance use. Medical and physical comorbidities are also somewhat common, including growth restriction, vision problems, hearing problems, congenital heart defects, and seizure disorders. Flake indicated that greater awareness of the associations between these conditions and FASD can help more clinicians consider FASD as a potential factor to aid in diagnosis and treatment.

**Fig. 2:**
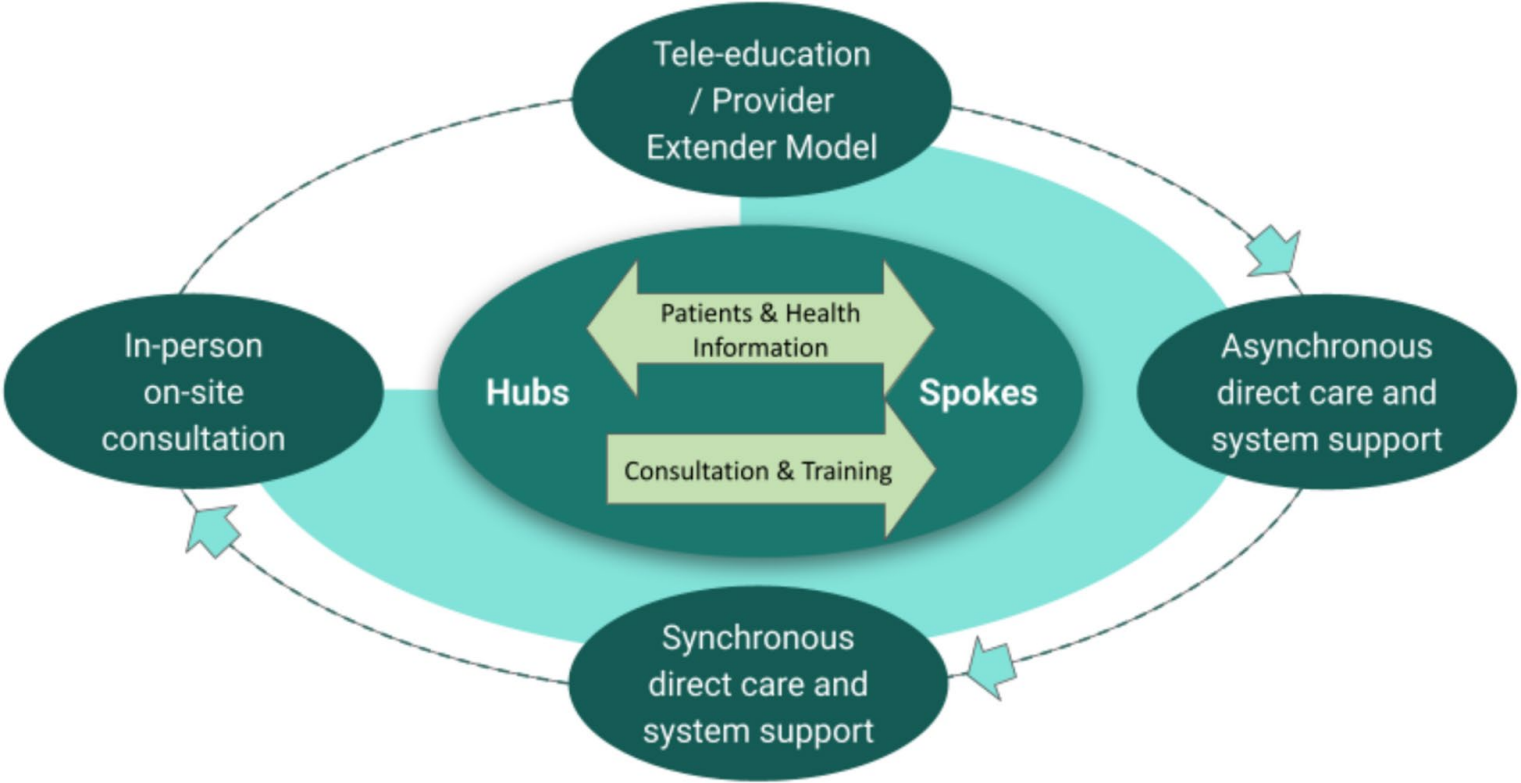
Avenues for interaction and support within a hub-and-spoke model for provider-to-provider consultation. Source [28]: Flake EM. (2025). Hub and Spoke Model in Provider to Provider Consultation: Connecting Tiers of Care for Military Children with Prenatal Alcohol Use [PowerPoint slides]. FASD Annual Workshop

Flake described how provider-to-provider consultation can help clinicians become more comfortable diagnosing neurodevelopmental disorders in general and FASD in particular. A hub-and-spoke model allows clinicians to interact with each other and with patients and families through channels that may be asynchronous or synchronous, can be conducted virtually or in-person, and can involve training, consultation, or care (Fig. 2).

Flake and colleagues are now in the early stages of piloting this hub-and-spoke model for FASD care in the Pacific Northwest, where there is a high concentration of military families but limited physical connectivity among medical providers and patients across different sites. Building on a similar network set up for ASD in this region, the team aims to extend the model to enhance care for the FASD community. They plan to evaluate how this model performs in terms of expanding access to care, improving providers’ experiences facilitating FASD diagnosis and services, and effectiveness in enhancing FASD pathways of care.

## Learning from living experiences

Jennifer Wisdahl, FASD United, introduced the living experience panel, which consisted of two individuals, Barbara (Barb) Clark and Emily Hargrove, who spoke about their experiences with FASD. Opening the panel discussion, Wisdahl noted that her own three children, who are young adults with FASD, have experienced multiple co-occurring conditions including hearing and vision problems, environmental and food allergies, mental health problems, and a wide variety of neurodevelopmental disorders. She added that her family has experienced barriers to care as a result of these co-occurring diagnoses.

Clark provides FASD training, coaching, and consulting for families and professionals and has multiple personal connections with FASD. One of her children, now 26 years old, has been diagnosed with FASD but she said that all five of her children were exposed to alcohol in utero and may also be affected. In addition, Clark has FASD herself, a diagnosis she received only recently as an adult.

Hargrove was diagnosed with FAS as an infant and has long served as an advocate for people in the FASD community, including as an inaugural member of the Adult Leadership Collaborative (ALC) of the FASD Changemakers. In addition, she is a PhD student studying FASD, bringing both an advocacy and research perspective to FASD.

The panelists explored the challenges they and their families have faced related to the multiple manifestations of FASD and its co-occurring conditions. To begin the discussion, Hargrove described a survey the ALC conducted to understand the range of health issues experienced by people with FASD. With responses from over 500 individuals, the study found that respondents faced an increased risk for 258 of 260 health issues that were included in the survey, and were more likely to develop these issues at younger ages, compared with the general population. These health conditions include a wide variety of issues such as heart conditions, hearing problems, cancers, cognitive issues, reproductive disorders, and a multitude of others.

When asked how this plethora of co-occurring conditions affects the ability of families to access and sustain support for children with FASD, Clark said that “it can be extremely difficult for families to navigate care systems to understand and address the issues their child is facing”. In the case of her daughter, who was diagnosed with FASD at age six, Clark said that a history of behavioral challenges led many clinicians to dismiss her child’s complaints, which led to delays in diagnosing a rare condition. In response to such experiences, Clark began providing training for clinicians, which she said has helped lead to improvements in FASD awareness and care.

Building on these points, Hargrove said that the most helpful thing clinicians can do is to believe their patients. Hargrove and Clark both commented that many physicians have dismissed them when they have brought up their FASD in the context of other health conditions. Doctors often assume there is no connection between FASD and conditions within their area of medical specialty, when in fact there might be. Another issue, Hargrove said, is that doctors may overly “dumb down” their communication style after a patient mentions their FASD, on the assumption that the patient lacks the intellectual capacity to understand complex information. Clark suggested that researchers can help to address these issues by focusing on elucidating the whole-body impacts of FASD, understanding co-occurring conditions, debunking stereotypes about cognitive capacity among people with FASD, and strengthening communication and training for medical, mental health, and other professionals that patients encounter as they navigate systems of care.

Wisdahl and Clark noted that while children with FASD can benefit from many types of services—from occupational therapy to physical therapy to appointments with various specialists—the sheer number of services needed and the frequency of appointments can combine to create a challenging lifestyle for a family. Clark said that becoming overly focused on seeking out every possible test or intervention can detract from the ability to simply spend time together and enjoy family life. It can also pathologize children when what they desire most is normalcy. As a family coach, she said she often advises families to choose one issue to focus on rather than trying to address everything at once, and to occasionally take a few months off from interventions to focus on connecting with their child. She added that taking a less intensive approach to addressing the unique needs of a child with FASD can also be beneficial for siblings and the family as a whole. In her own family, she appreciated how one medical professional would always ask how the other children in her family were doing to ensure they were adequately supported in addition to addressing the more intensive needs of one child. She added that taking care to avoid shaming families is also essential for clinicians to help support families holistically.

From a clinical care perspective, panelists also emphasized the importance of coordinated multidisciplinary care, which can lead to more harmonized and holistic care as opposed to cycling through each specialist individually. As an added bonus, this approach can also help to reduce the time children and families spend visiting specialists and receiving services, allowing children more time to engage in meaningful activities like sports, extracurriculars, and hanging out with friends. Hargrove added that cost can be an important consideration and barrier to accessing care, which she experienced personally growing up. She underscored the importance of ensuring families affected by FASD are able to access the services their children need.

In addition to opportunities to improve care pathways during childhood, the panelists discussed some additional issues that people with FASD can face as they grow older. Speaking to concerns around reproductive health, Hargrove noted that her own pregnancy experience was quite positive, as many of her health challenges lessened in severity during pregnancy. However, there is a broader question about whether people with FASD may have a higher risk of passing along genetic conditions to their children. While she has not experienced fear or worries about that related to her own pregnancies, Hargrove said it is important for clinicians to be compassionate when caring for women who may have these fears. Looking forward, Hargrove and Clark also suggested particular topics for further research, including the connection between FASD and cysts and potential effects on accelerated aging.

## Reflections

Tracey Pérez Koehlmoos and Tom Donaldson closed the workshop by thanking attendees and speakers for sharing their insights and supporting candid discussions. They commented that attendees—whether from the military, research organizations, advocacy groups, or other backgrounds—undoubtedly came away from the event feeling more empowered to move forward in their efforts to improve research, care, and support for people with FASD and their families.

## Data Availability

All data is contained within the workshop report.

## Appendix A

### Workshop attendees

**Table:**

Organization Type	Organization	Name
Government	CHSR-USUHS	Teresa Russell
	CHSR-USUHS	Jade Parker
	CHSR-USUHS	Tracey Koehlmoos
	Frederick County MD Task Force	Shannon Kaisler
	Uniformed Services University	Elizabeth Lee
Health and support service providers	Children's Service Center	Summer Krochta
	Dream Acres/Kansas FASD Support Network	Kathryn Meinhardt
	FASCETS Center for Neurodiversity	Stacey Chart
	Formed Families Forward	Kelly Henderson
	Formed Families Forward	Stacia Stribling
	Fusion Center Network and University of Arkansas for Medical Sciences	Elizabeth Cleveland
	Institute for Health and Recovery	Reuben Kittrell
	KS, TX & FASCETS	Julia Rivera
	MassFASD, Institute for Health and Recovery Inc.	Kristen Eriksen
	Orchids FASD Services Wisconsin, Inc.	Guida Brown
	Papillion Center for FASD	Tim Diffenderfer
	Papillion Center for FASD	Chris Troutt
	Papillion Center for FASD- Tennessee	Joshua Legg
	Proof Alliance	Kendra Gludt
	The Florida Center	Tamra Cajo
	The Florida Center for Early Childhood	Jennifer Werden
Medical and research organizations	American Academy of Pediatrics	Rosa Arvizu
	American Academy of Pediatrics	Hope Barrett
	Boston University School of Public Health	Rachel Adams
	Henry M. Jackson Foundation	Madison Cirillo
	Henry M. Jackson Foundation	Eric Flake
	Henry M. Jackson Foundation	Ameryth Hargrove
	Henry M. Jackson Foundation	Michelle Mellers
	Henry M. Jackson Foundation	Sharon Pritchett
	Henry M. Jackson Foundation	Ilse Rivera
	Henry M. Jackson Foundation	Zoe Solomon
	Henry M. Jackson Foundation/USU	Kyle Apilado
	LSU Health Sciences Center, New Orleans	Amy Ward
	Mt. Hope Family Center, University of Rochester	Christie Petrenko
	Oak Ridge Associated Universities	Katherine Chyka
	Seattle Childrens	Heather Carmichael Olson
	Texas A&M College of Medicine	Siara Rouzer
	Texas A&M University	Amanda Mahnke
	University of Alaska, Anchorage	Lily Bastian
	University of Florida	Nicole Marlow
	University of Rochester, Mount Hope Family Center	Julianne Myers
	UW Institute on Development and Disability	Misty Pruner
	Weill Cornell Medicine	Omar Rahman
Resource networks and non-profits	AFMC	Shaneca Smith
	Arkansas None for Nine	David Deere
	Arkansas None for Nine	Amy Smith
	Arkansas None for Nine	Lewis Walker
	Children and Family Futures	Sidney Gardner
	Families Rising	Jamole Callahan
	Families Rising	Barb Clark
	FASD - North Dakota	Carl Young
	FASD Action Group Hawaii	Darlyn Chen Scovell
	FASD Collaborative Project	Angelle Gremillion
	FASD Collaborative Project	Emily Rusnak
	FASD Focus NW	Crystal Bauer
	FASD Focus NW	Debbie England
	FASD Network of Northern California	Rachel Sing
	FASD Network of Southern California	Annette Kunzman
	FASD Texas Network	Hannah Ross
	FASD United	Emma Baldwin
	FASD United	Sarah Brown
	FASD United	Susan Carlson
	FASD United	Terri Cullen
	FASD United	Tom Donaldson
	FASD United	Olivia Gargiulo
	FASD United	Casidee Gonzales
	FASD United	Andy Kachor
	FASD United	Chris Melfi
	FASD United	Sophie Sissi
	FASD United	Kathryn White
	FASD United	Jennifer Wisdahl
	Fostering Forward Michigan	Lara Bouse
	Hawaiʻi FASD Action Group	Amanda Luning
	Illuminate Colorado	Lex Loutzenhiser
	Kansas FASD Support Network	Holly Helus
	NC FASD Informed	Allison Parker
	NC FASD Informed	Holly Warren
	NCFASD Informed, Inc	Kathy Hotelling
	Texas FASD Network	Rebecca Ciesielski
	Texas FASD Network	Debra Ross
Companies and consultancies	Clover Leaf Wealth Strategies LLC	Cortney Heykoop
	KRBR Consulting LLC	Kate Boyce Reeder
Organization not specified		Naghum Alfulaij
		Michael Anderson
		Alissa Brasfield
		Konstance Causey
		Jean Clarkson
		Whitney Emerson
		Lindsay Goeckeritz
		Emily Hargrove
		PJ King
		Liz McHugh
		Karen Poteet
		Janet Thomas
		Jude Warren
		Malette Young

## Abbreviations

FASD: Fetal alcohol spectrum disorders
DoW: Department of War
MHS: Military Health System
USUHS: Uniformed Services University of the Health Sciences
CHSR: Center for Health Services Research
HSR: Health Services Research
PAE: Prenatal Alcohol Exposure
LoEs: Lines of Effort
FAS: Fetal Alcohol Syndrome
SWYC: Survey of Well-Being of Young Children
ID: Intellectual disabilities
ASD: Autism spectrum disorder
ADHD: Attention-deficit/hyperactivity disorder
INROADS: Intersecting Research on Opioid and Alcohol Misuse, Addiction, and Disability Services
ALC: Adult Leadership Collaborative
